# Wellbeing and occupational risk perception among health care workers: a multicenter study in Morocco and France

**DOI:** 10.1186/s12995-016-0110-0

**Published:** 2016-05-04

**Authors:** Doina Ileana Giurgiu, Christine Jeoffrion, Christine Roland-Lévy, Benjamin Grasset, Brigitte Keriven Dessomme, Leila Moret, Yves Roquelaure, Alain Caubet, Christian Verger, Chakib El Houssine Laraqui, Pierre Lombrail, Christian Geraut, Dominique Tripodi

**Affiliations:** Department of Occupational Medicine and Environment Health, HCWs Research Laboratory, 5 rue du doyen Boquien, Nantes University Hospital, Nantes, 44 093 France; “Lucian Blaga” University of Sibiu, 10 Victoriei Boulevard, Sibiu, 550024 Romania; Psychology Laboratory of Pays de la Loire (LPPL - UPRES EA 4638), Department of Psychology, University of Nantes, BP 81 227 44312 Nantes cedex 3, France; Cognition, Health, Socialization, EA6291 University of Reims Champagne-Ardenne, 57, rue Pierre-Taittinger, Reims, 51 096 France; Department of Public Health, Nantes University Hospital, 35 rue Saint Jacques, Nantes, 44 000 France; Laboratory of Ergonomics Epidemiology Health and Work, LEEST-UA InVS - IFR 132- UPRES EA 4336, University of Angers, Faculty of Medicine, University Hospital, 4 rue Larrey, 49933 Angers Cedex, France; Occupational Medicine Department, 6 rue Henri Le Guilloux, University Hospital of Rennes, Rennes, 35 000 France; Graduate School of Health Engineering and Project Management, 24 rue Lafontaine, Quartier Racine, Casablanca, 20 100 Morocco; Public Health Department, SMBH, Paris 13 University, 74 avenue Marcel Cachin, Bobigny, 93017 France

**Keywords:** Occupational stress, Risk exposure, High strain, Public hospital, Medication use

## Abstract

**Background:**

The study analyzes health care workers’ (HCWs) occupational risk perception and compares exposure to occupational risk factors in Moroccan and French hospitals.

**Method:**

Across nine public hospitals from three Moroccan regions (north, center and south), a 49 item French questionnaire, based on the Job Content Questionnaire, and 4 occupational risks subscales, was distributed to 4746 HCWs. Internal consistency of the study was determined for each subscale. Confirmatory factor analysis was conducted on the Moroccan questionnaire. Psychosocial job demand, job decision latitude and social support scores analysis was used to isolate high strain jobs. Occupational risks and high strain perception correlation were analyzed by univariate and multivariate logistic regression. A comparative analysis between Moroccan and French (Nantes Hospitals) investigations data was performed.

**Results:**

In Morocco, 2863 HCWs (60 %) answered the questionnaire (54 % women; mean age 40 years; mean work seniority 11 years; 24 % physicians; 45 % nurses). 44 % Moroccan HCWs are at high strain. Casablanca region (1.75 OR; CI: 1.34–2.28), north Morocco (1.66 OR; CI: 1.27–2.17), midwives (2.35 OR; 95 % CI 1.51–3.68), nursing aides (1.80 OR; 95 % CI: 1.09–2.95), full-time employment (1.34 OR; 95 % CI 1.06–1.68); hypnotics, sedatives use (1.48 OR; 95 % CI 1.19–1.83), analgesics use (1.40 OR; 95 % CI 1.18–1.65) were statistically associated to high strain. 44% Moroccan HCWs are at high strain versus 37 % French (Nantes) HCWs (*p <* 0.001).

**Conclusion:**

Moroccan HCWs have high strain activity. Moroccan HCWs and more Moroccan physicians are at high strain than Nantes HCWs. Moroccan and French’s results showed that full time workers, midwives, workers using hypnotics, and analgesics are at high strain. Our findings underscore out the importance of implementing a risk prevention plan and even a hospital reform. Further research, with an enlarged study pool will provide more information on psychosocial risks (PSR) and HCWs’ health.

## Background

The workplace is now regarded as a key player in the origin of various illnesses and as a determinant of the individual’s well-being, along with the individual’s life-style. In recent years occupational health, particularly occupational mental health, has been the subject of particular attention. Health at work has become a major concern in our society and a priority in public health [1]. Among numerous risks found in the workplace, one group, covering various risks, has lately emerged as a major public health concern and a challenge to the occupational health research field: the psychosocial risks (PSR). PSR are not just another type of risk factors, but one to be found at the center of the complex architecture of work conditions.

The French Ministry of Work and Employment (2013) describes the concept of PSR as “risks to health, mental as well as physical, created at least by work through social and psychological mechanisms”. PSR include stress, internal violence and external violence (INRS^1^). Internal violence involves physical or verbal abuse, bullying, intimidation, and conflicts with colleagues or managers; it includes situations of moral and sexual harassment. People in the workplace produce PSR towards others. External violence is carried out towards employees, in the working context, by people from outside the organization it-self (users, families, providers and suppliers, customers, consumers, etc.). The most exposed workstations are those in contact with the public, specifically when dealing with people in precarious situations or having psychological problems.

It seems necessary to distinguish the concept of “risk”, i.e. the probability of being confronted to risk, thus creating “risk factors”, from “disorder”, which is a consequence of exposure to risk. A large number of studies, in the medical sector and particularly in the hospital context [2–6], have shown the importance of PSR in terms of consequences on both physical and mental health. These studies show that the influence of negative organizational climate on nurses’ health [7], along with interactions between physical and PSR factors which can generate musculoskeletal disorders [8]. The impact of occupational safety climate in hospitals confirms the link between PSR and workers’ health [9]. In Europe, according to recent work conditions surveys, the influence of psychosocial risk factors in the workplace ranked second in the recent major developments hierarchy [10]. The European Agency for Safety and Health at Work (EU-OSHA), in its report on emerging risks [11] indicated that there is a strong correlation between eight variables (size of establishment; whether the establishment is a part of a larger entity; sector (public or private); gender composition of establishment’s workforce; age; composition of establishment’s workforce; proportion of foreigners in establishment’s workforce; sector of activity; country) considered for inclusion in the Occupational Safety Health composite score. An imbalance between the number of industrial accidents and the number of occupational illnesses has also been noted: even if the first is decreasing, the latter is increasing [12]. In France, indicators defined by experts enable us to demonstrate that the workplace PSR factors often contribute to the occurrence of health problems, such as cardiovascular diseases, mental health disorders or musculoskeletal disorders [13].

As the workforce in the European Union (EU) is ageing and the work intensity is constantly increasing, questions on steps to be taken to keep the workforce active have arisen along with the need for a well-designed policy [14]. The over 40 million people in the EU suffering from consequences of work-related stress, which translates into over 20 billion € of health and absenteeism costs [15], contributes to underlining the economic and social importance of addressing the issue of work-related PSR.

The guidelines of the European Commission (EC) on work-related stress itemized the steps to be taken, of which the first was identifying the risk, with its sources and consequences, by the means of monitoring “job content, working conditions, terms of employment, social relations at work, health, well-being and productivity” [16]. One of the important methods for detecting work-related stress, i/e. PSR, is to use a risk-perception evaluation; the individual’s subjective experience will be the generator of symptoms and diseases, going from transient increase in heart rate and blood pressure to developing cardiovascular diseases, and from brief anxiety or depression to serious mental health problems. The EC, in its *Improving quality and productivity at work. Community strategy 2007–2012 on health and safety at work,* has identified a significant proportion of employees who acknowledged the impact work has on their health: “35% of workers on average feel that their job puts their health at risk” [17].

Psycho-social strain is not limited to European workplaces. However, intercontinental comparisons of stress in the workplace are rare. Among these few studies, this study compares Morocco and France, which is quite interesting as labour laws in Morocco are close to French laws. Furthermore, in hospitals, the work organization is similar in both countries. Therefore, in this paper, we analyse the Moroccan situation and compare it to the French one. Currently, international comparative studies on occupational risks in the public hospitals are scarce and only few researchers concentrated on psychosocial stress in Moroccan HCWs [18–22].

Even though Karasek’s JCQ was translated, tested and used in many different countries [23–30], none focused on Moroccan HCWs’ PSR perception. Studies in health care concentrate mainly on nurses, probably because it is a rather homogenous group that is quite easy to access, contrary to more sensitive groups such as physicians, who are a complex target. In fact, some studies even excluded physicians because of the difficulty in obtaining a significant number of responses [27]. As Robert Karasek has already stated in his 1998 study on psychosocial job assessment through JCQ [28], one of the problems with demanding jobs holders is their reluctance to participate in research projects.

The aim of the present study is twofold: (i) to assess risk perception among Moroccan HCWs, including physicians, using a validated questionnaire; and (ii) to make an analysis of exposure to occupational risk factors variance in Moroccan public hospitals. Special attention was given to the different aspects which influence the occurrence of “high strain” situations: mainly ergonomics and overall working conditions. The study aimed at making a comparative evaluation of perceived risks in the healthcare sector of Morocco and France, by analyzing comparable structures. The main goal was to find out if, in comparable structures from different cultures, with similar occupational categories, stress was perceived in the same manner or not. The concluding goal was to develop a model for high strain perception to be of use for crafting and implementing a specific prevention plan.

## Methods

The study was a cross-sectional multicenter investigation, conducted in French and Moroccan public hospitals, in which labor laws, as well as the work organization, are very similar.

Moroccan public hospitals were selected from the three Moroccan regions: north, center and south. On Morocco, the north region hospitals were those of Kenitra, Oujda and Larache; the southern region hospitals were those of Agadir and Marrakech. The center region or the Casablanca region covered Baouafi, Sekkat, Settat and Khouribga’s hospitals. The Moroccan Public Health Ministry’s authorization was obtained before beginning the investigation.

The same investigation was conducted in three French public hospitals, covering all departments through the same self-administered questionnaire.

The study population is composed of all Health Care Workers (HCWs). The activity sectors were Anesthesia and Intensive Care, Biology, Oncology, Digestive, Emergency, Geriatric Care, Medicine, Mother and Child Hospital, Nephrology, Urology and Transplant, Neuroscience, Dentistry, Orthopedics, Physiotherapy and Occupational therapy, Psychiatry, Public Health and Occupational Medicine, Head and Neck, Imaging, Research, Teaching, and the Chest department.

A steering committee, involving the university hospital management, engineers and public health and occupational medicine staff created a self-administrated questionnaire targeting occupational hazards and the main psychological factors encountered. The workplace health and safety committee (WPHSC), in agreement with the French labor law, approved the study protocol.

The questionnaire includes 49 questions grouped in four subscales. The first 29 items on work and psychosocial relations were extracted from Karasek’s Job Content Questionnaire (JCQ) [23] and were validated in France by Niedhammer [26]. The next items stemmed from the French study, with authorization of use provided by the authors [24, 25]: eight items targeted workplace ergonomics; three items addressed workplace environment risk. The last 8 items focused on passive smoking, alcoholism and medication use (hypnotics, sedatives and analgesics) and were taken from the French National Institute of Prevention and Health Education’s “Adults Health Barometer”. The first cluster corresponded to the main variable, which is the psychosocial risk factor. The two other clusters were cofactors for the analysis of risks related to ergonomic, biomechanical, organizational, environmental, toxic and addictive factors (French version of CAGE questionnaire for alcoholism screening).

The questionnaire was distributed and collected upon completion by Moroccan medical students who first informed the personnel of its purpose, items and anonymity. All 4 746 employees from the nine public hospitals were considered eligible.

Data capture was made by students, by means of an Excel mask designed by the Medical Evaluation and Therapeutic Education Office of the Medical Information and Public Health Evaluation Department of Nantes University Hospital. Data was analyzed with SAS/STAT 8.2 and SPSS 13.0. Questionnaires with missing answers were excluded from the analysis. Descriptive analysis of data was made: percentage of answers for qualitative variables, mean and Standard Deviation for quantitative variables. Job Decision Latitude (JDL), Psychological Job Demand (PJD) and Social Support (SoSu) scores were calculated according to Karasek et al. [23] and Niedhammer et al. [26]. Threshold values for high PJD, low JDL and low SoSu were respectively set to 24, 72 and 22 [26]. High strain occupations had PJD score over 24, JDL score under 72 and SoSu score under 22.

Categorical variables correlations (e.g., between medication use and alcohol consumption) were analyzed by chi-squared tests. Quantitative correlations were analyzed by Student’s *t*-tests. Other qualitative variables correlations (e.g., between scores and age) were estimated by Pearson’s correlation coefficient. Factors influencing the scores were determined by one-way ANOVA and by MANOVA for occupational categories adjustment. Step-by-step logistic regression and multivariate analysis was used for identifying factors connected to high PJD associated with low JDL. A comparative analysis of Moroccan and French (Nantes Hospitals) investigative data was performed. The studies in Morocco and France were carried out a few months apart. Moroccan hospitals have the same ward structure and occupational categories as French hospitals. The France data was extracted from the 2012 published study [25] with permission from authors. Confirmatory factor analysis was conducted on the Moroccan questionnaire.

## Results

### Descriptive data

In Morocco, 2,863 HCWs (60 % of the total hospital staff) answered the questionnaire, of whom 54 % were women. The mean age of the sample was 40.4 years [SD ± 10.2]. The mean work seniority was 11.3 years [SD ± 9.9]. 97 % of HCWs had a permanent employment contract and 79 % worked full-time. One should note thatin Morocco many HCWs have a second job because of the low salary they receive. The participation rate varied among the different hospitals, with leading scores from Larache (North Morocco) and Sekkat (Casablanca) hospitals (94 % and 91 %) and the lowest score (38 %) from Marrakech hospital (South Morocco). The participation rate was higher in the administration departments (67 %) and lower in surgery and technical-medical departments (57 %). The technical-medical personnel had the highest participation rate (74 %), followed by the janitors and the administrative personnel (73 %, 70 %), with the technical staff (27 %), the psychologists (33 %) and the nursing assistants (37 %) having least participated in the study.

### Questionnaire’ validity

Cronbach alpha coefficients for the French and Moroccan models are presented in Table 1.

**Table 1:** Internal consistency of the French and Moroccan questionnaire

Cronbach’s alpha coefficients	French questionnaire	French questionnaire submitted to
	submitted to French HCWs	Moroccan HCWs
Job Decision Latitude scale	0.771	0.596
Psychological Demand scale	0.713	0.3832^a^
Social Support scale	0.814	0.764

^a^Cronbach’s alpha for PJD was clearly low; and after confirmatory factorial analysis, excluding the two inversed questions of JCQ, it was adjusted at 0.490

Internal consistency of the study was determined for each subscale; in the Moroccan study, Cronbach’s alpha coefficients were 0.596 for JDL, 0.383 PJD, 0.764 for SoSu, 0.737 for workplace ergonomics, 0.450 for environmental risks and 0.516 for medication use. Cronbach’s alpha for PJD was clearly low, and after confirmatory factorial analysis, excluding two inversed question of JCQ, it was adjusted at 0.490. This low score was probably due to a poor understanding of the French nuances. PJD and SoSu scores were not correlated (*r* = -0.025, NS). JDL and PJD scores were weakly correlated (*r* = 0.110, *p <* .01), as well as JDL and SoSu scores (*r* = 0.248; *p <* 0.01).

### Psychosocial risks

JDL, PJD and SoSu scores were diverse among occupational categories (*p <* 0.001) (Table 2).

**Table 2:** Moroccan-French decision latitude and psychological demand scales

Psycological demand scale France (Nantes)				Psychological demand scale Morocco			
Occupational category	Mean*	N	SD	Occupational category	Mean*	N	SD
Psychologist	21,5	17	2,8	Psychologist	23.0	2	0
Janitor	22,3	59	3,9	Rehabilitation personnel	23.1	39	3.2
Technical-medical personnel	22,7	116	3,0	Nursing assistant	23.3	39	2.7
Technical staff	23,4	92	3,2	Administrative	23.6	299	3.4
Other	23,5	30	3,9	Social/Educational staff	24.0	16	3.5
Social/Educational staff	23,8	26	3,7	Other	24.1	53	2.7
Nursing-assistant	24,4	237	3,4	Technical staff	24.2	27	2.8
Rehabilitation personnel	24,7	32	3,6	Specialist nurse	24.2	463	3.3
Administrative	24,7	222	3,7	Technical-medical personnel	24.4	88	3.4
Midewife	24,8	20	2,1	Janitor	24.6	60	2.5
Nurse	24,8	103	3,2	Nurse	24.7	717	3.2
Physician	25,3	204	3,4	Physician	25.0	630	3.3
Non specialist nurse	25,6	355	3,5	Care management	25.2	26	2.8
Care Management	25,9	127	3,4	Midwife	25.8	148	3.1
Total	24,6	1640	3,6	Total	24.6	2607	3.3
Decision Latitude scale France (Nantes)				Decision Latitude scale Morocco			
Occupational category	Mean	N	SD	Occupational category	Mean	N	SD
Janitor	61,4	59	11,4	Technical staff	53.9	25	10.4
Nurse-assistant	63,6	235	8,8	Other	62.1	58	9.3
Technical-medical personnel	66,4	114	9,2	Administrative	62.9	297	9.9
Administrative	67,9	223	11,2	Social/Educational staff	62.9	13	10.4
Non specialist nurse	68,6	359	8,7	Janitor	64.3	60	13.8
Nurse	69,1	109	8,3	Nursing assistant	64.4	41	6.8
Technical staff	70,3	91	11,1	Technical-medical personnel	65.1	88	10.6
Psychologist	72,7	17	19,5	Care management	65.4	27	7.8
Other	73,1	30	11,6	Midwife	65.6	142	9.9
Social/Educational staff	74,1	27	7,5	Nurse	66.2	729	9.4
Midewife	74,3	20	9,0	Specialist nurse	66.9	461	8.8
Rehabilitation personnel	75,2	36	5,9	Physician	69.5	630	9.0
Care management	76,3	128	8,0	Rehabilitation personnel	74.5	38	9.9
Physician	77,1	203	8,4	Psychologist	83.0	2	9.9
Total	69,6*	1651	10,4	Total	66.5*	2611	9.8

*SD* Standard Deviation

*p < 0.001

**Fig. 1:**
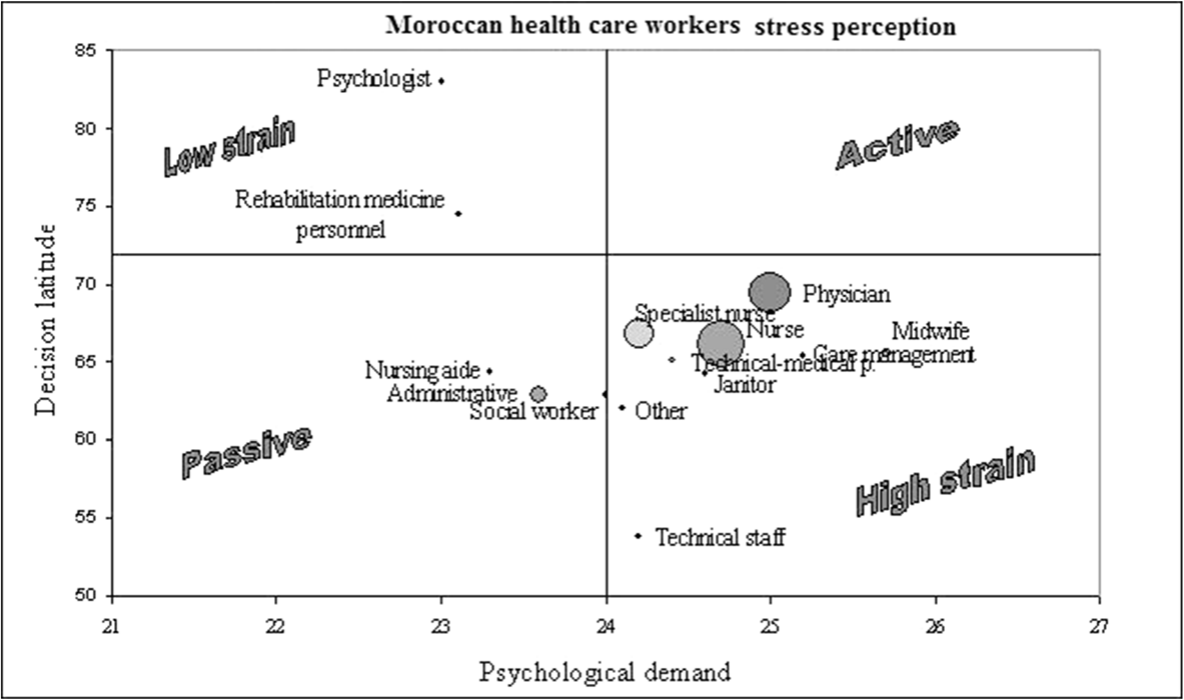
Moroccan Health care workers stress perception

Moroccan results: the respondents with the highest risk (High strain: occupational categories with the riskier combination of low job decision latitude, high psychological demand and low social support) were technical staff and care management personnel, but physicians, nurses and specialized nurses also had a high risk (Table 3). No occupational category placed itself in the active job quadrant—high demand and high control (Fig. 1).

**Table 3:** Comparative analysis of Moroccan-French high strain and iso strain

	Moroccan HCWs *N =* 2863	Nantes HCWs *N =* 1612	*p*
High PJD	62 %	66 %	< 0.01
Low JDL	76 %	62 %	< 0.001
High Strain (high PJD, low JDL)	44 %	37 %	< 0.001
Iso Strain (high PJD, low JDL, low SoSu)	25 %^a^	19 %^b^	< 0.001
Decision Latitude scale comparison			
Region	Mean	*N*	SD
North Morroco	64,8	821	9,7
Center Morocco	65,4	862	9,4
South Morocco	68,8	947	9,7
France (Nantes)	69,5	1661	10,4
Total	67,6	4291	10,2
Psychological demand scale comparison			
Region	Mean	*N*	SD
South Morocco	24,42	933	3,38
North Morocco	24,59	816	3,59
Center Morocco	24,62	879	2,80
France (Nantes)	25,27	1640	4,01
Total	24,82	4268	3,59
	Nantes *n =* 1612	Morocco *n =* 2863	*p*
Decision latitude scale	69,5 +/- 10,5	66,4 +/- 9,8	*
Psychological demand scale	25,2 +/- 4,0	24,5 +/- 3,3	*

*SD* Standard Deviation

*p < 0.001

^a^technical staff, care management

^b^nurse assistant, technical-medical personnel, non specialist nurse

**Fig. 2:**
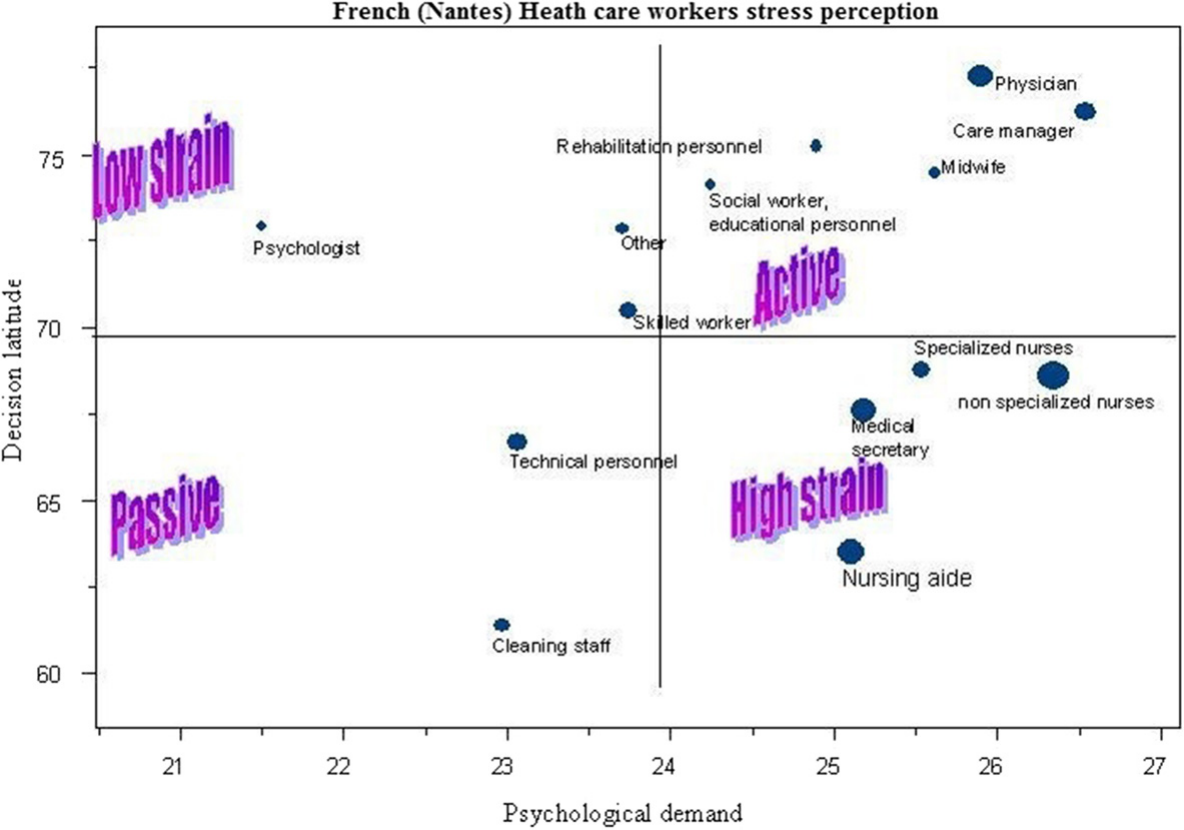
French (Nantes) Health care stress perception

JDL, PJD and SoSu were dependent of gender (adjusted OR = 1.26, Table 4)… Temporary employees had higher social support (*p <* 0.001). Full-time employees scored lower in JDL and SoSu (*p <* 0.001), but higher in PJD (*p <* 0.001). 20 % of the respondents reported using hypnotics or sedatives on a fairly regular basis (more than once during the week preceding the investigation), with an equal proportion of men and women. These respondents were older (43.2 years [SD ± 9.9] versus 40.3 years [SD ± 10.0], *p <* 0.001). 41 % of the respondents reported taking analgesics fairly regularly (more than once during the week preceding the investigation). Among them, more were women (43 % women versus 39 % men, *p <* 0.05) and they were older (41.9 [SD ± 10.1 years] versus 40.3 [SD ± 9.9 years], *p <* 0.001). Use of hypnotics or sedatives was significantly correlated to low JDL (*p <* 0.001). It was also correlated to a high PJD (*p <* 0.05) and to a lower SoSu (*p <* 0.01). Use of analgesic could be explained by high prevalence of musculoskeletal disorders. Use of hypnotics could be explained first by a high level of strain and second by sleeping disorders. Alcohol intake investigation did not show significant results, as the survey was incidentally carried out in the month of Ramadan, when, in accordance with a religious principle, the use of alcohol is prohibited, which implies that even if some participants were taking alcohol, they would not mention it. After adjustment and multivariate analysis, high strain perception was strongly correlated with north and center morocco hospitals, sex (males are more at risk), employment contract and type of contract (full time HCWs are more at risk), occupational category (midwifes), workplace environment (bad accessibility, noise, brightness at workstation), the use of hypnotics and sedatives (see Table 4). Moroccan—French hospitals comparison (Fig. 1, Fig. 2, Table 3, Table 5**).** Comparative analysis of Moroccan and French (Nantes) investigations showed that 76 % of the Moroccan HCWs and 62 % of the Nantes University Hospital HCWs experienced low JDL situations (*p <* 0.001). High strain situations (low JDL and high PJD) were recorded for 44 % of Moroccan HCWs, versus 37 % of Nantes University Hospital HCWs (*p <* 0.001). Adding social isolation, the high strain quadrant grouped another 25 % of Moroccan HCWs, opposed to the 19 % of Nantes HCWs (*p <* 0.001). In order to analyze high strain in all HCWs, the HCWs Moroccan and French groups were merged. The risk factors which were strongly associated with a problematic context (high PJD and low JDL), in univariate and multivariate analysis of the merged Moroccan and Nantes groups, were the Moroccan group, with a higher strain in Northern Morocco and the Casablanca region, especially among midwives and nursing aides, more with full-time employment with a greater seniority in workplace, which includes those who use hypnotics and analgesics (see Table 5).

**Table 4:** High Strain and occupational risk factors in Moroccan HCWs, univariate and multivariate analysis

Problematic context =	high strain	*n*	%	Crude OR	Crude CI	Adjusted OR	CI
Group *n* = 2472	Casablanca region (control)	387	50	1		1	
	North Morocco	399	51	0.62	0.50–0.75	1.03	0.82–1.31
	South Morocco	353	38	1.05	0.86–1.29	0.73	0.56–0.96
Gender	Female	620	48	1		1	
*n* =2453	Male	512	45	0.89	0.76–1.05	1.26	1.04–1.53
Employment Contract	Permanent	1096	46	1		-	-
*n* =2462	Temporary	37	48	1.09	0.67–1.75		
Employment type	Full-time	923	49	1.68	1.37–2.05	1.65	1.24–2.19
*n* =2448	Part-time	201	36	1		1	
Occupational category *n* =2452	Administrative	123	45	1		1	
	Care management	16	62	1.94	0.80–4.79	2.10	0.85–5.23
	Specialist nurse	181	42	0.87	0.63–1.20	0.79	0.56–1.12
	Nurse	335	49	1.15	0.86–1.53	1.07	0.77–1.48
	Nursing assistant	16	46	1.02	0.48–2.18	0.96	0.42–2.21
	Janitor	27	45	0.99	0.54–1.80	0.95	0.51–1.78
	Midwife	94	69	2.71	1.72–4.29	2.33	1.41–3.85
	Rehabilitation medicine personnel	7	18	0.27	0.11–0.68	0.24	0.09–0.62
	Social worker	6	50	1.21	0.34–4.37	1.34	0.36–5.08
	Physician	244	41	0.84	0.62–1.13	0.65	0.47–0.91
	Technical-medical personnel	45	54	1.43	0.85–2.42	1.33	0.75–2.34
	Technical staff	12	50	1.21	0.49–3.00	0.44	0.15–1.31
	Other	21	45	0.98	0.50–1.90	1.12	0.52–2.42
Accessibility in the workplace *n* = 2504	Satisfactory	750	42	1		1	
	Unsatisfactory	363	52	1.50	1.26–1.78	1.24	1.01–1.53
Comfortable work posture *n* = 2423	Satisfactory	379	41	1		-	-
	Unsatisfactory	698	47	1.30	1.10–1.53		
Work plan height *n* = 2266	Satisfactory	417	41	1		-	-
	Unsatisfactory	587	47	1.31	1.11–1.55	-	-
Manual handling *n* = 2136	Easy	489	43	1		-	-
	Difficult	495	49	1.27	1.07–1.51	-	-
Noise level *n* = 2520	Correct	563	39	1		1	
	Incorrect	556	51	1.65	1.40–1.93	1.31	1.08–1.60
Lighting or brightness level *n* = 2537	Correct	501	38	1		1	
	Incorrect	624	52	1.76	1.50–2.06	1.45	1.19–1.76
Ambient temperature level *n* = 2547	Acceptable	409	40	1		-	-
	Unacceptable	721	48	1.38	1.17–1.62		
Exposure to hazardous chemicals *n* = 2324	Satisfactory	279	47	1		-	-
	Unsatisfactory	792	46	0.96	0.79–1.16		
Exposure to radiation *n* = 2330	Satisfactory	185	43	1		-	-
	Unsatisfactory	881	47	1.17	0.94–1.45		
Safety instructions *n* = 2383	Present or available	287	40	1		-	-
	Absent or unavailable	777	47	1.29	1.08–1.54	-	-
Use of hypnotics or sedatives *n* =2384	Yes	250	53	1.50	1.22–1.85	1.41	1.11–1.79
	No	811	43	1		1	
Use of analgesics *n* =2413	Yes	499	50	1.36	1.15–1.61	1.37	1.13–1.66
	No	587	42	1		1	

*CI* Confidence Interval

*OR* Odds Ratio

**Table 5:** High Strain in Moroccan and Nantes HCWs, multivariate analysis

Problematic context = High strain		*n*	%	Adjusted OR	CI
Group	Nantes	606	39	1	
*n* = 4011	North Morocco	399	51	1.66	1.27–2.17
	South Morocco	353	38	1.07	0.83–1.38
	Casablanca region	387	50	1.75	1.34–2.28
Employment type	Full-time	1295	46	1.34	1.06–1.68
*n = 3717*	Part-time	331	37	1	
Occupational category	Administrative	208	43	1	
*n* = 3984	Care management	48	33	0.64	0.35–1.16
	Specialist nurse	232	44	0.81	0.56–1.09
	Nurse	513	50	1.03	0.78–1.35
	Nursing assistant	129	52	1.80	1.09–2.95
	Janitor	48	43	0.87	0.52–1.45
	Midwife	104	67	2.35	1.51–3.68
	Rehabilitation medicine personnel	10	15	0.26	0.12–0.55
	Social worker	13	35	0.77	0.28–2.14
	Physician	287	36	0.70	0.53–0.94
	Technical staff	32	29	0.52	0.28–0.97
	Other	27	36	0.91	0.47–1.75
Seniority *n* = 3095		–	–	1.02	1.01–1.04
Use of hypnotics	Yes	315	53	1.48	1.19–1.83
*n* = 3799	No	1342	42	1	
Use of analgesics	Yes	691	49	1.40	1.18–1.65
*n* = 3835	No	991	41	1	

*CI* Confidence Interval *OR* Odds Ratio

## Discussion

The goal of the present study was to assess risk perception among Moroccan HCWs with a validated questionnaire, in order to make an analysis of exposure in relation to occupational risk factors variance in Moroccan public hospitals, and to compare the results with French HCWs (Nantes). The Labor Law in Marocco and the work organization on public hospitals are very similar to French ones which allowed meaningful comparisons of data over time.

The first strength of this investigation is the size and diversity of the HCWs’ group, with a 60 % participation rate in the health care sector and a 67 % response rate by physicians constituting trustworthy data for analysis. The second originality of this paper is that it compares two cultures, French and Moroccan participants, knowing that we did not find any study of the kind comparing participants from Morocco.

The data show that significantly more Moroccan HCWs are exposed to high strain than Nantes Hospital HCWs. North Morocco and Casablanca region HCWs are subjected to higher strain than both South Morocco and French HCWs. Whereas the French study found many active jobs: physician, care management, midwife, the Moroccan study brought to attention a crowded high-strain quadrant in the JDL-PJD diagram (Fig. 1), which included all of the above occupations, alongside nurses and even janitors, which were passive category in the French survey. On the contrary, none of the Moroccan HCW jobs qualified as active. There might be a cultural mark involved, but the main reasons were economic and organizational: Moroccan physician and nurse staff appeared to be clearly insufficient in number; the doctors’ activity was always intense and they complained of suffering from excessive time pressure and the technical equipment was often inadequate, outdated or faulty. Moreover, the staff was poorly paid and had a low degree of job independence, which led to the fact that many HCWs had to have a second job because of low pay.

Among the main perceived occupational risks, HCWs also indicated aggression, additional workload, musculoskeletal disorders and stress. Though physicians and nurses were considered as holding and active jobs [23], there are many investigations with opposite results. Columbian nurses had high demands but also high control [24]. The 2004 French report [31] on public hospitals HCWs showed an high level of job autonomy for physicians, but a much lower one for nurses and nursing assistants. The 2011 Italian study on risk factors in healthcare professionals [32] showed low JDL and SoSu scores for ancillary workers, but no significant variation in PJD, which is consistent with our findings. But differences in job demands are not unusual between countries and cultures. Pisanti et al. [33] brought evidence of higher pressure among Italian nurses, as opposed to Dutch nurses, which is also consistent with the differences between PJD scores for our French and Moroccan nurses. But there are many elements that can influence job perception and thus job satisfaction: the difficult climate of today’s economic crisis leaves its mark on job security, even if all three scores of PJD, JDL and SoSu are high enough to place the nurses in the active jobs category [29].

High strain among nurses is also not uncommon and the reasons outlined by researchers mainly reside in gender disparity: women report lower levels of decision latitude [23]. Nevertheless, no gender influence on high-strain scores was found here. However there are grounds for high strain in women: cultural elements, tradition, or increased workload (based on unclear competencies) forced on nurses [34]. Moroccan women have a specific social status: Moroccan traditional family structure gives them the household responsibilities, which may in turn increase their general stress level [35]. Social status and high or low level of education can also influence the understanding of any questionnaire content and purpose, and may affect answer, bringing in bias [36].

As already mentioned, this is the first cross-sectional multicenter study to assess PSR perception and to estimate occupational stress in Moroccan non-university public hospitals. It showed a good overall participation rate and a very strong willingness of hospital HCWs to participate in the survey (94 % participation rate in Sekkat Casablanca and 91 % participation rate in Larache); we believe this speaks in itself about both the awareness and the need for changes in working conditions. Besides evaluating, another study purpose was to build ground base for organizational and work environment changes, to decrease occupational stress and to increase job control and social support. It has already been shown that, among many consequences, high JDL and SoSu enhanced safety participation [37]. This may not be the case yet for our study group, which has unsatisfying levels of JDL for the majority of HCWs, but constitutes another trigger for policy design. If an intervention plan was to be implemented in all Moroccan public hospitals, once started, an evaluative re-analysis of PSR would be not only necessary but highly useful [38].

Our study showed no positive correlation between age and high strain (0.97 adjusted OR, CI: 0.96–0.99), similar to results from larger and more heterogeneous groups [23], though JDL and SoSu perception decreased with age, which is similar to the Nantes study results [19]. But, we must consider the risk of cardiovascular disease (CVD) that comes with age, one which is already linked to job strain [39]. In a meta-analysis of 14 prospective cohort studies, Kivimäki et al. (2006) suggested an average 50 % excess risk of coronary heart disease when occupational stress was present [40]. A connection between occupational stress and inflammation appears to exist, the latter triggering CVDs [41].

Since we found no explanation for temporary HCWs having higher SoSu and for senior workers having lower SoSu, there is need for further investigation here. Full-time employees experienced more stress than part-time employees, which was consistent with findings in the Nantes’ study. There was certainly more pressure in full-time contract and the perceived responsibility increased for these. Longer working time enhanced a greater workload and more stress, as its imprints on scores showed: lower JDL (65.5 [SD ± 9.6]), lower SS (21.6 [SD ± 3.9]) and higher PJD 24.7 [SD ± 3.3].

Moroccan HCWs took significantly more hypnotics and sedatives (20 %) and more analgesics (40 %) than Nantes HCWs (9 % and 25 %); the use of medication was significantly associated to high strain (*p <* .001). Medication use in Moroccan HCWs proved to be much higher than other reports from hospital environments. Virtanen et al. conducted a study in 21 Finnish hospitals and found lower usage rate: 5 % for anxiolytic and hypnotic medication and 16 % for pain-killers [42]. Use of sedatives and hypnotics is certainly linked to alternative shifts, night work, stress, workload and fatigue. Analgesics use was associated to musculoskeletal disorders, even if the declared average weight lifted by Moroccan HCWs [51.1kg SD ± 109.1 kg] was much lower than the average weight lifted by Nantes HCWs [130.7 kg SD ± 255.9 kg]. However we can question the use of such drugs which presupposes often the presence of psychological and/or psychiatric disorders in these subjects and we regret not having evaluated these people with some specific tests for the psychiatric screening. Further studies should have recourse to such tests. Alcohol intake in Moroccan HCWs could not be compared to that of Nantes HCWs, because of the alcohol prohibition during the Ramadan month, which we had not taken into account when designing the investigation time schedule, therefore leading to a limitation.

Though PJD was lower in Moroccan HCWs, it still held a high score, and when associated to low JDL it concentrated a higher percentage of high strain HCWs (44 %) than the ones reported in other studies: 16.5 % in Taiwanese HCWs [28], 25 % in European workers [43]. This raises high concern and it calls for an immediate intervention. As for the different occupational categories, it must be noted that Moroccan physicians were at a higher level of strain than physicians from any other country. In our study they were not considered an active occupation. Work overload, insufficient staffing, poor doctor-patient relationship, low hospital budget are all triggers of occupation status corrosion. All studies on perceived stress focusing on or including doctors have placed them in the high demand-high control job category. This indicates a higher risk degree in Moroccan doctors for developing CVDs and mental disorders. Higher depression rates than in general population have been reported in physicians with high work demands [44]. On the other hand, Moroccan rehabilitation medicine personnel had passive jobs, similar to other reports [44, 45], but unlike its Nantes counterpart, which found itself in the active quadrant of Karasek’s JCQ model. This proved a much lower degree of independence.

Our study has encountered a number of limitations. The weak points of self-reporting must first be restated. There is no solution to avoid or limit individual variation in PSR perception. In the Moroccan study, Cronbach’s alpha for PJD was clearly low, and after factorial analysis, excluding two inversed question of JCQ, it was adjusted at 0.490. This low score was probably due to a poor comprehension of French language. The target is personal, subjective sensation and understanding and not objective quantification. But there is sufficient evidence that perception is at the origin of changes in well-being and of ill health, and their prevention is occupational medicine’s ultimate goal. Participants in the study have not been randomly selected. Reasons for unresponsiveness were not requested. Results may have been influenced by exclusion of participants due to missing item answers. Karasek’s demand-control model leaves out the effort-reward balance and the patient-caregiver relationship, and there is no data on absenteeism, presence, patient admission rates, factors to be taken into account in future investigation. The study did not take into account the patients/staff ratio and did not investigate differences in staff norming between the analyzed healthcare structures in Morocco and France. The questionnaire version used in Morocco wasn’t perfectly identical to that used in the Nantes study, since some workstation ergonomics items weren’t identical and therefore could not be mentioned. However, the study provides an accurate picture of PSR in Moroccan public hospitals. It brings forward a map of stressful occupations and it points out priorities for action.

## Conclusions

This paper is innovative by comparing hospital staff in two different countries which were not compared before. More Moroccan HCWs in general and more Moroccan physicians are at high strain than Nantes’ HCWs and physicians. Use of hypnotics and analgesics is at high level in Moroccan HCWs. Full time workers, midwives and workers using hypnotics, sedatives and analgesics are under high strain. Our results provide elements for improving the working conditions and well-being of health care workers in Morocco. They stress out the importance of implementing a risk prevention plan and even a hospital reform. Further research, with an enlarged study pool and a more exhaustive analysis, will bring more information on PSR and HCWs’ health.

## Footnotes

1 Institut National de Recherche et de Sécurité/National Institute of Research and Security http://www.inrs.fr/
